# Development and Evaluation of a Mobile Decision Support System for Hypertension Management in the Primary Care Setting in Brazil: Mixed-Methods Field Study on Usability, Feasibility, and Utility

**DOI:** 10.2196/mhealth.9869

**Published:** 2019-03-25

**Authors:** Daniel Vitório Silveira, Milena Soriano Marcolino, Elaine Leandro Machado, Camila Gonçalves Ferreira, Maria Beatriz Moreira Alkmim, Elmiro Santos Resende, Bárbara Couto Carvalho, André Pires Antunes, Antonio Luiz Pinho Ribeiro

**Affiliations:** 1 Telehealth Center Hospital das Clínicas Universidade Federal de Minas Gerais Belo Horizonte Brazil; 2 Post-Graduate Program in Infectious Diseases and Tropical Medicine Faculdade de Medicina Universidade Federal de Minas Gerais Belo Horizonte Brazil; 3 Telehealth Network of Minas Gerais Belo Horizonte Brazil; 4 Medical School Universidade Federal de Uberlândia Uberlândia Brazil; 5 Medical School Universidade Estadual de Montes Claros Montes Claros Brazil

**Keywords:** telemedicine, clinical decision support system, cardiovascular disease, hypertension

## Abstract

**Background:**

Despite being an important cardiovascular risk factor, hypertension has low control levels worldwide. Computerized clinical decision support systems (CDSSs) might be effective in reducing blood pressure with a potential impact in reducing cardiovascular risk.

**Objective:**

The goal of the research was to evaluate the feasibility, usability, and utility of a CDSS, TeleHAS (tele–*hipertensão arterial sistêmica*, or arterial hypertension system), in the care of patients with hypertension in the context of a primary care setting in a middle-income country.

**Methods:**

The TeleHAS app consists of a platform integrating clinical and laboratory data on a particular patient, from which it performs cardiovascular risk calculation and provides evidence-based recommendations derived from Brazilian and international guidelines for the management of hypertension and cardiovascular risk. Ten family physicians from different primary care units in the city of Montes Claros, Brazil, were randomly selected to use the CDSS for the care of hypertensive patients for 6 months. After 3 and 6 months, the feasibility, usability, and utility of the CDSS in the routine care of the health team was evaluated through a standardized questionnaire and semistructured interviews.

**Results:**

Throughout the study, clinicians registered 535 patients with hypertension, at an average of 1.24 consultations per patient. Women accounted for 80% (8/10) of participant doctors, median age was 31.5 years (interquartile range 27 to 59 years). As for feasibility, 100% of medical users claimed it was possible to use the app in the primary care setting, and for 80% (8/10) of them it was easy to incorporate its use into the daily routine and home visits. Nevertheless, 70% (7/10) of physicians claimed that the time taken to fill out the CDSS causes significant delays in service. Clinicians evaluated TeleHAS as good (8/10, 80% of users), with easy completion and friendly interface (10/10, 100%) and the potential to improve patients’ treatment (10/10, 100%). A total of 90% (9/10) of physicians had access to new knowledge about cardiovascular risk and hypertension through the app recommendations and found it useful to promote prevention and optimize treatment.

**Conclusions:**

In this study, a CDSS developed to assist the management of patients with hypertension was feasible in the context of a primary health care setting in a middle-income country, with good user satisfaction and the potential to improve adherence to evidence-based practices.

## Introduction

Hypertension is a major modifiable cardiovascular risk factor responsible for substantial morbidity and mortality worldwide. It causes around 9.4 million deaths every year [1]. According to the World Health Organization, the prevalence of this condition in adults older than 25 years is 29.2% in men and 24.8% in women [2], which results in a global prevalence of over 1 billion people. Despite this high prevalence, the blood pressure control levels are as low as 30% of treated patients worldwide [3].

In the last three decades, clinical practice guidelines addressing hypertension management invariably recommended that cardiovascular risk assessment must be a core feature of hypertension care [4-7]. Brazilian guidelines also recommend the systematic assessment of cardiovascular risk during hypertension management and the use of statins if needed [8]. Cardiovascular and cerebrovascular diseases are the leading causes of death in low- and middle-income countries (LMIC) [1], including Brazil [9], and are responsible for the increasing use of health system resources, quite often from preventable complications. Despite Brazilian Society of Cardiology recommendations on the use of multiple risk scores to assess cardiovascular risk and advocacy of the use of a global risk assessment based in the 10-year Framingham heart score risk [8,10], Brazilian physician adherence to its use is poor [11].

To face these challenges, the health systems in LMIC struggle to improve their quality and overcome underfunding and lack of resources [12]. In Brazil, a large country with a decentralized health system, primary care physicians have limited access to point-of-care information resources that could assist clinical decision making and improve quality of care. In this context, mobile health (mHealth) technologies might be used to ease the access of health programs to a large number of individuals at relatively low cost [13-16]. When integrated to clinical decision support systems (CDSSs), mHealth technologies might increase the accuracy of diagnosis and treatment [12]. However, most studies were performed in high-income countries, implying that mHealth is still at an early stage of development in low-income countries [13,14,17].

Most of the literature that evaluated CDSSs in the management of hypertension focused on outcomes, with mixed results [18-22]. Despite the recommendation of international and Brazilian medical societies, few CDSSs addressing hypertension included the assessment of cardiovascular risk and few studies in LMIC evaluated what characteristics were responsible for implementation failure or success or addressed feasibility and user satisfaction in the primary care setting [23,24]. No study evaluated the use of a CDSS in the care of patients in Brazil. Thus, we conducted this study to develop a CDSS that integrates cardiovascular risk assessment, monitoring of blood pressure, nonpharmacological measures, and guidance to drug prescription. We also aimed to test the feasibility of implementing it in Brazilian primary care units as well as to assess its usability and utility, identifying facilities and barriers to its use.

## Methods

### Design of the Study

This study was conducted in four steps, according to the Medical Research Council framework [25].

#### Identify Gaps in Usual Care Through Literature Review

We assessed epidemiological studies and systematic reviews on hypertension management as well as feasibility studies, randomized controlled trials, and systematic reviews on CDSSs to evaluate which gaps were already known. Additionally, the topic was discussed in meetings with Brazilian health system stakeholders and primary care physicians to identify other issues.

#### Identify Components of the Intervention Through Discussion With Experts

Meetings and internal workshops were conducted to discuss the topic with primary care physicians. Primary care physicians, internal medicine specialists, cardiologists, and endocrinologists discussed the gaps with experts in information technology to identify solutions and components of an intervention.

#### Clinical Decision Support System Development

The CDSS, named TeleHAS (tele–*hipertensão arterial sistêmica*, or arterial hypertension system), was developed based in clinical practice guideline recommendations. Brazilian and international guidelines assessing hypertension as a main subject or in the context of other comorbidities such as diabetes or chronic renal disease [4,5,26-31] were reviewed, and clinical rules for hypertension and cardiovascular risk management were derived and organized into a decision tree (Multimedia Appendix 1). In case of conflicting recommendations, the one with the best level of evidence, most compelling recommendation, or latest publication was chosen. When a guideline recommendation was not available or was considered outdated, rules based on best scientific evidence were used, including evidence-based summaries, synopses, or syntheses. The decision tree was then organized as a CDSS on a modular basis and installed on a tablet using the Android 4.1 operating system.

**Figure 1:**
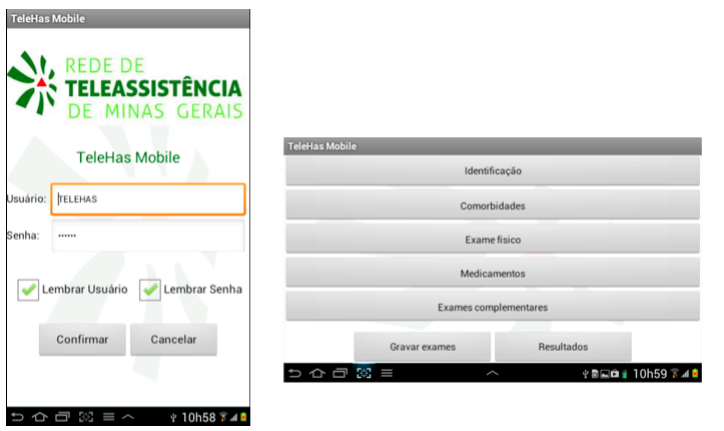
TeleHAS open screen and main menu.

**Figure 2:**
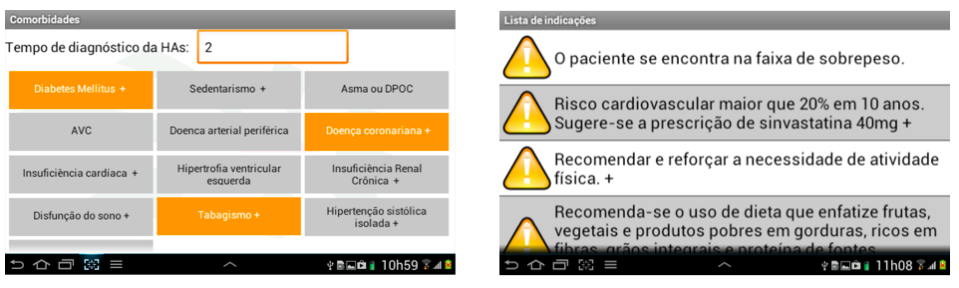
TeleHAS previous diseases and alerts menu.

The CDSS consists of a structured clinical evaluation assessing identification, medical history, physical examination, current medications, and laboratory and image test results (Textbox 1). Only the patient name and date of birth were considered mandatory fields. The interface was developed to be intuitive and self-explanatory. Data requested were manually entered and included variables of interest in the management of hypertensive patients: blood pressure, lipid profile, renal function tests, microalbuminuria or proteinuria, liver enzyme tests, and electrocardiogram, among others. Body mass index, estimated glomerular filtration rate using the Cockcroft-Gault formula [32], and cardiovascular risk based on the Framingham score [33] were calculated using the data entered and displayed immediately on screen. After data are entered, the CDSS presents suggestions and recommendations about pharmacologic and nonpharmacologic interventions including physical activity, diet recommendations, and medication dosages and interactions (Figures 1 and 2).

After patient registration, any subsequent consultation was recorded in the database in a file under the patient name. Data were recorded in TeleHAS and transmitted to a telehealth care central whenever internet connection was available. The CDSS was updated if problems were identified. A Web-based data panel was developed to help researchers access the database.

The CDSS was electronically tested to verify that recommendation results matched the prespecified decision tree. Manual insertion of data by a physician was later performed to verify the recommendation response suitability. After adjustments, TeleHAS was submitted for analysis to an expert panel comprising 2 cardiologists and 3 primary care physicians, known as technical reference, for a period of 7 days. Structured questionnaires and semistructured interviews were used to assess strengths, inconsistencies, and satisfaction with the device use. The participants were asked to classify their general impression of the CDSS as very appropriate, appropriate, indifferent, or inappropriate. It was then readjusted with the necessary changes according to the criticisms and suggestions from the expert panel.

**The five main menus of TeleHAS.**

- Identification: patient name, date of birth, mother’s name, and sex
- Comorbidities: may be used to select other diseases or risk factors presented by the patient
- Physical exam: blood pressure, waist circumference, weight, and height
- Laboratory studies: lipid profile, biochemical panel, echocardiography, and electrocardiography data
- Medications: several blood pressure medications categorized by class

#### Evaluate Feasibility Through Field Study

The field study was conducted in Montes Claros, the largest city in the north of the Minas Gerais state (population 361,000), 270 miles north of the state’s capital. It has a human development index of 0.77 [34], slightly above the Brazilian average. The municipal primary care system is composed of 88 primary health centers in urban and rural areas with health teams composed by one physician and one nurse and a variable number of community health workers. Despite the existence of a university and two medical schools, clinicians have limited access to specialist referral and continuing education due to the distance to the country’s main centers.

All 66 primary care physicians from Montes Claros were invited to attend a lecture on hypertension diagnosis and management. Of the 63 attendees, 51 agreed to join the study and 10 were randomly selected. The selected doctors used TeleHAS in routine care of hypertensive patients to build up their impressions about the CDSS for 6 months. Physicians planned and individualized the frequency of blood pressure measurements for each patient.

A nurse and an information technology technician held biweekly visits to address issues and resolve difficulties in the CDSS use and allow data transfer to the TeleHAS network server through a 3G connection available only on the nurse’s tablet due to its high cost in Brazil.

**Figure 3:**
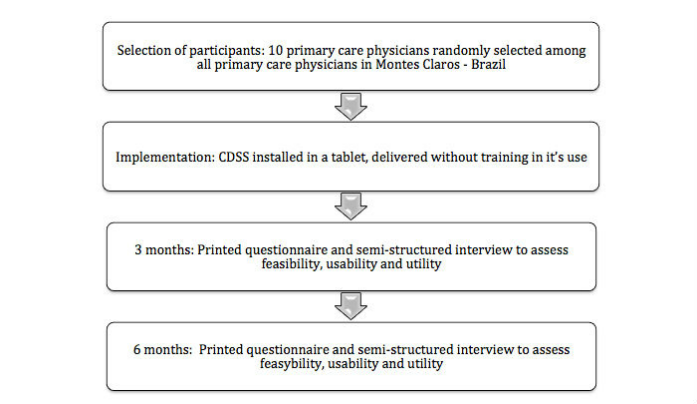
Study design.

Paper-based questionnaires evaluating feasibility, usability, and utility of the CDSS were developed and applied with semistructured interviews at the end of 3 and 6 months (Figure 3). Perceived feasibility, usability, and utility questions were rated on a Likert scale from 1 (strongly disagree) to 5 (strongly agree) [35]. The final score was defined as the mean of the scores for all of the questions.

### Data Analysis

Categorical variables were presented as frequencies and proportions. As the sample size of physicians was small, continuous variables were expressed as median and interquartile range. Data analyses were performed using SPSS Statistics for Windows version 21.0 (IBM Corp).

### Ethics Statement

The study was approved by the ethics committee of the Universidade Federal de Minas Gerais, Minas Gerais, Brazil. Informed, written consent was obtained from all participants contributing data to the study.

## Results

### Expert Panel

All specialists considered the initial menu, identification, comorbidities, medicine, and complementary exam screens as adequate (Table 1). Doctors unanimously considered the TeleHAS interface, alert content, appearance, and speed of alert generation adequate. They generally reported that TeleHAS used the best available evidence and suggested changes based on literature or use of alternative guidelines when needed. The suggested changes were incorporated in the software.

Specialists perceived TeleHAS as an intervention that could change clinical practice.

The CDSS helps in the process of decision making for treatment of patients with hypertension, both in pharmacologic and nonpharmacologic therapy.

Family physician

I believe it can be very helpful to control high blood pressure.

Cardiologist

There was a unanimous impression that the device has the potential to deliver updated information to health care providers and education promotion is a major feature of its use. It was highlighted that the device has the potential to promote implementation of evidence-based recommendations.

Great potential to promote best scientific evidence implementation for the individual patient.

Family physician

When asked about the feasibility of TeleHAS in the clinical setting, all participants agreed it was feasible.

TeleHAS has the potential to improve clinical practice quality, and it’s applicable to the routine of primary care services.

Family physician

One primary care physician considered the physical exam screen inadequate. He stated that he would like to enter data about other conditions not associated with hypertension or cardiovascular risk such as glycemic control and data from skin, respiratory, and abdomen semiology. He suggested that a free-text field could be added to address this demand.

### Field Study

Participant characteristics are described in Table 2. Age varied from 27 to 59 years (median 31 years). The median length of professional experience in a primary care setting was 2.5 years (ranging from 0 to 20 years) and time working in the municipality was 1.5 years.

During the 6 months of field study, participant physicians registered 535 patients in the TeleHAS database and performed 632 consultations.

### Feasibility, Usability, and Utility

Quantitative analysis of impressions for feasibility, usability, and utility is described in Tables 3-5.

Although doctors were motivated to use TeleHAS, finding the time to enter data was a major concern. Physicians believed it caused significant delay in the daily routine and developed alternative forms of use to deal with this challenge. Some used the CDSS in educational groups and on days specifically scheduled to attend patients with hypertension. Others used it daily when attending a patient with hypertension. Due to the scarce time available, some of them attended patients without the CDSS and filled it out later, saving remarks for the next appointment. In the case of one clinician, this choice was due to fear of patient opinion.

...I was concerned that they might find I was distracted or writing something else, like sending messages during the consultation.

Clinician

Work duplication was also identified as a problem, and clinicians demanded a printed handout to deliver to patients and attach to the patient record at the end of the consultation.

**Table 1:** Expert panel perception of TeleHAS for each screen (n=5).

Screen	Rating		
	Very adequate, n (%)	Adequate, n (%)	Inadequate, n (%)
Initial menu	3 (60)	2(40)	0 (0)
Identification	3 (60)	2 (40)	0 (0)
Comorbidities	1 (20)	4 (80)	0 (0)
Physical exam	0 (0)	4 (80)	1 (20)
Medicine	2 (40)	3 (60)	0 (0)
Complementary exams	1 (20)	4 (80)	0 (0)

**Table 2:** Characteristics of primary care physicians in the study (n=10).

Variable		Value, n (%)
Sex, female		8 (80)
**Time since graduation (years)**		
	<5	7 (70)
	5-10	1 (10)
	>10	2 (20)
**Specialty**		
	Angiology	1 (10)
	Family medicine	2 (20)
	Geriatrics	1 (10)
	Occupational medicine	1 (10)
	Pediatrics	1 (10)
	None^a^	4 (40)
**Self-reported knowledge of information technology**		
	Inadequate	2 (20)
	Satisfactory	4 (40)
	Good	3 (30)
	Excellent	1 (10)
Use of any form of technology before TeleHAS, yes		9 (90)
Computer available in the workplace for routine use, yes		0 (0)
Internet access in the workplace, yes		5 (50)
**Internet use frequency**		
	Daily	9 (90)
	Monthly	1 (10)
Completed continuing education on management of hypertension or cardiovascular risk in the last year, yes		6 (60)
**Major sources of continuing education**		
	Guidelines	3 (30)
	Books	2 (20)
	Articles	1 (10)
	Congress	1 (10)

^a^Residency is not a prerequisite for doctors who work in primary care in Brazil.

**Table 3:** Feasibility score on a 5-point scale by item (n=10).

Item	Score mean
The CDSS^a^ can be used in the primary care setting.	4.5
It can be used in home visits.	4.2
It is easy to incorporate in work routine.	4.0
Internet connection is not essential for the use of the CDSS.	3.6
The CDSS does not cause significant delays in daily routine.	3.0

^a^CDSS: clinical decision support system.

**Table 4:** Usability score on a 5-point scale by item (n=10).

Item	Score mean
My overall evaluation of the CDSS^a^ is good.	4.5
The CDSS screens are easy to understand.	4.5
The definitions of comorbidities are clear and unambiguous.	4.1
The CDSS fields are easy to complete.	4.3
The CDSS is intuitive and requires no previous training to use.	3.4
The CDSS is stable, and no errors occur during use.	3.0

^a^CDSS: clinical decision support system.

**Table 5:** Utility score on a 5-point scale by item (n=10).

Item	Score mean
I believe that the CDSS^a^ might improve the treatment of hypertensive patients.	4.5
Reading the recommendations of the CDSS, I had access to new information on hypertension and cardiovascular risk.	4.1
According to my previous knowledge, I believe the recommendations generated by the CDSS are appropriate.	4.1
The CDSS was useful to calculate the cardiovascular risk of hypertensive patients.	3.8
The CDSS was useful to promote cardiovascular disease prevention actions among my patients.	4.3
The CDSS helped me treat my patients.	4.6
I used the recommendations to modify the behavior of my patients.	4.0
I would recommend the CDSS to my colleagues.	4.7

^a^CDSS: clinical decision support system.

With regard to usability, during the interviews, all physicians classified the identification screen as practical and simple to use. Clinicians also reported the comorbidities screen as simple and intelligible.

Although TeleHAS was designed to be intuitive, some physicians believed training was essential for its proper use, even though clinicians did explore its features and learned the hidden tasks it was designed to do (eg, the majority found out that date of birth could be inserted by the keyboard or by a cursor displayed after continuously pressing the data field).

Most clinicians stated that other chronic conditions should be addressed in the CDSS and requested the option to enter glucose levels, diabetes mellitus complications such as diabetic foot, and other classes of medication for associated conditions such as methimazole, insulin, metformin, isosorbide and cilostazol.

Some clinicians observed errors during CDSS use. The most discussed one was that the software would sometimes stop working. This was due to a fault in the Android system that would stop functioning if the tablet enter key was pressed twice. No primary health care center had Wi-Fi connection available. System actualizations and data transfer to the TeleHAS database server occurred whenever Wi-Fi connection was available, at the physician’s home or during the visit by the nurse and information technology technician. Eventually, due to the absence of 3G coverage or inadequate connection speed, the nurse needed to set up an appointment with the physician in the city to proceed with data transfer.

With regard to utility, physicians reported that TeleHAS eventually changed clinical practice. Some of them changed their blood pressure measurement techniques by reading the description available in the CDSS. Others started to measure blood pressure in 3 positions to complete all the fields available in TeleHAS.

For the physical exam screen, clinicians suggested a change in the sequence available in the CDSS, as they normally make the first measure with the patient seated.

Sometimes I entered the measure in the wrong field, because I start with the patient seated. Then I had to enter the measures again, after other measures.

Family physician

It was perceivable that clinicians worked with different levels of systematization. Only three of them calculated cardiovascular risk systematically using the 10-year global risk assessment score chart. Physicians perceived TeleHAS as helpful in promoting cardiovascular risk assessment and prevention actions.

Some clinicians perceived the CDSS fulfillment and recommendations delivered as repetitive.

After a while, you get tired of reading the same things. You don’t have much time available [to deal with the repetitive content].

Clinician

However, some considered the repetition a CDSS strength.

Sometimes we remember to guide the patient only about medication, and we forget to talk about nonpharmacologic treatment. The alerts are repetitive, but it helps us to remember.

Family physician

I used to forget to ask about the salt in their diet. With the CDSS, I remembered to ask about it with every patient.

Geriatrics

The accuracy of the CDSS cardiovascular risk calculation was considered unsatisfactory by 30% (3/10) of participants. In the interviews, this issue was often discussed. The clinicians reported that the cardiovascular risk calculator sometimes presented spurious results and made them confused about the correct estimated risk. This error was found to occur when the date of birth was not entered or entered incorrectly.

## Discussion

### Principal Findings

This field study designed to evaluate the feasibility and usability of a CDSS to assist hypertension and cardiovascular risk management found that it is feasible to implement such technology in a primary care setting in a middle-income country with good satisfaction by health professionals. Equally important, the study elicited strengths and faults of the CDSS and helped identify facilities and barriers for TeleHAS implementation.

In Brazil, primary care physicians are commonly young and inexperienced doctors, frequently recently graduated [36,37], and in this study the majority of participants were women with less than 5 years of professional experience after graduation. Participant clinicians’ profiles were variable regarding clinical experience and expertise, clinical specialty, intimacy with information technology, and forms of knowledge update. Primary care physicians often used the CDSS in their work routine and inserted a large amount of data in the TeleHAS database. They reported having learned new skills and knowledge through TeleHAS, felt comfortable using its guidance to change their practice and make clinical decisions, and rated the CDSS as having good usability and usefulness for knowledge dissemination, with potential influence for better hypertension management.

This feasibility study showed several barriers for the implementation of TeleHAS, including health professional issues, telecommunication structure, and health system infrastructure. Physician resistance to adopting information technologies is one of the main barriers for implementation, often due to poor satisfaction with physical characteristics of digital technologies, difficulties with their use, and low effectiveness of devices [38]. In our TeleHAS evaluation, clinicians reported good satisfaction with the CDSS, and it is presumable that this will facilitate its use in large scale. In this sample, the previous use of information technology was low. Clinicians did have some difficulties managing the software and tablet, and although it did not deeply affect the use of the CDSS, some physicians had the perception that training would be needed for TeleHAS use. More effort should be made to improve the CDSS to make it as intuitive as possible for better scalability.

With regard to the telecommunication structure, problems with internet connection, poor broadband coverage, and high cost of 3G limited data transfer to the TeleHAS database server. Problems in telecommunication structures that limit dissemination of mHealth services in LMIC are similar to those seen in LMIC medical care, like services limited in scope, unevenly distributed across geographic areas, and of variable quality when available [39]. This issue was mitigated because TeleHAS was designed to function without an internet connection, but it is still required for data transmission and system update. Support by government policies to improve the quality of telecommunication structure is important to avoid future greater limitations for TeleHAS use.

Barriers to CDSS use were also identified. Low computerization of the primary health centers was a major limitation for the CDSS implementation. There was a perceptible demand for communication technologies in primary health system and much of the demand presented by physicians was related to the need to register and organize patient records and clinical data. This is in agreement with CDSS usability tests performed previously. In a study to assess the usability of a CDSS for the management of diabetes, the absence of electronic medical records was also pointed out as a major barrier for system definitive implementation in primary care [40]. Combination of CDSS with electronic health records, communication technologies integration in the structure of health care, and progressive computerization of primary health centers should stimulate use of these technologies.

Another major barrier for TeleHAS use in primary care was health professionals’ excessive workload. The work duplication generated by the need to register data in the tablet and in the patient records limited the use of the CDSS. In a meta-analysis of 311 unique studies on CDSSs, including 148 randomized controlled trials, several features have shown to be predictors of improved health care process measures, including integration with charting or order entry system with no need for additional clinician data entry [41]. Although TeleHAS was developed to be fully integrated into primary health care routine, its use as part of clinician workflow must improve to avoid work duplication. This was not possible at that time due to the fact that all primary care units, just like the majority of primary care units in Brazil, still use paper-based patient records. In the expert panel, a primary care physician suggested a free-text field to allow data entry of other conditions. As it was not our goal to substitute the paper-based patient records at that time, we opted not to make the change, but we included it in an updated version of TeleHAS. Despite the work duplication that we could not avoid, clinicians reported that it was possible to use the CDSS in their work routine and managed to find alternative ways to use it. A feasibility study of an mHealth app to assist nurses in the management of hypertension also found that professionals with heavy workloads felt the app demanded work duplication and was an extra burden in an already increasing workload [23]. Integration of the CDSS with the electronic health record in the primary care workflow may improve usability and reduce health professionals’ workload issues, enhancing its impact in clinical practice.

Task shifting to other health professionals is proposed as a solution to improve quality of care in chronic diseases. CDSSs designed to integrate the work of health professionals have been developed and may be the way for TeleHAS to diminish data entry requirements by clinicians. In a study performed in India, physicians and community health workers used a CDSS designed to address hypertension and assess cardiovascular risk. It was observed that such technology might aid to standardize the care and empower other health workers to help in cardiovascular risk assessment and patient care [24]. Care in primary health centers is unequal, as clinicians work with different strategies and environments and have different levels of knowledge and clinical experience. Despite the cardiovascular risk calculation in TeleHAS having errors and confusing some of the clinicians, the majority found the CDSS useful to calculate and manage the patients’ cardiovascular risk. This was probably because not all physicians performed systematic cardiovascular risk assessment before TeleHAS use, and this became part of their routine practice after they started using the CDSS. By asking for standardized data entry and providing repeated reminders about health care, mHealth technologies such as TeleHAS may help in health care standardization.

This study was designed to test and improve a CDSS and used qualitative assessments rather than clinical outcomes. However, its findings are very important, as usability testing is an essential step before software implementation in clinical practice or testing the impact in clinical outcomes. Poor usability may lead to various undesirable effects, ranging from user dissatisfaction and failed implementations to endangered patient safety [42]. This step was essential to test if TeleHAS was properly designed, assess its acceptance by the physicians, identify the necessity of improvements, and strengthen the CDSS for better adaptation to face the challenges for its implementation in clinical practice in order to obtain better results in blood pressure control and clinical outcomes. Data from individual patients were not addressed, so it is not possible to analyze the characteristics of the population in which the CDSS was applied or assess the impression of patients on the CDSS use by a clinician. The CDSS was improved, with optimization in the design of the screens and cardiovascular risk assessment and increased participation of health care practitioners other than doctors. The alerts were updated and information on diabetes control was added. Its impact on hypertension and diabetes control is being assessed in a large-scale study in 39 primary care units. Usability assessment will be performed with all health care practitioners in order to have a more representative sample of Brazilian primary care professionals.

### Conclusions

In this study, a CDSS developed to assist in decision making in hypertension and cardiovascular risk assessment was feasible in the context of the primary care setting, with good user satisfaction and possible positive impact on the implementation of guidelines, recommendations, and best available evidence. The study provided the opportunity to strengthen TeleHAS to face the barriers identified in its implementation. The CDSS is currently being tested in a large-scale clinical study to access clinical end points and the possibility to scale up its use for the primary care setting within the country and in other LMIC.

## Appendix

Multimedia Appendix 1 Example of TeleHAS decision tree.

## Abbreviations

CDSS: clinical decision support system
CNPq: Conselho Nacional de Desenvolvimento Científico e Tecnológico
FAPEMIG: Fundação de Amparo à Pesquisa de Minas Gerais
FINEP: Financiadora de Estudos e Projetos
LMIC: low- and middle-income countries
mHealth: mobile health
TeleHAS: tele–hipertensão arterial sistêmica (arterial hypertension system)
