# Supplementary material for: Development and Evaluation of a Mobile Decision Support System for Hypertension Management in the Primary Care Setting in Brazil: Mixed-Methods Field Study on Usability, Feasibility, and Utility
Source: JMIR Mhealth Uhealth. 2019 Mar 25;7(3):e9869. doi: 10.2196/mhealth.9869 (PMC6452279; doi:10.2196/mhealth.9869)
Supplement: Multimedia Appendix 1 [file mhealth_v7i3e9869_app1.pdf]

| | | | |
|---|---|---|---|
| 1. | 10-year Cardiovascular Risk > 20%. The prescription of simvastatin 40mg is suggested. | Cardiovascular risk> 20% AND Coronary disease = NO AND Stroke=NO AND Peripheral Artery disease =NO AND Heart infarction=NO AND REVASCULARIZATION=NO AND Glomerular filtration rate <45ml/Kg/min=NO AND Simvastatin=no AND Rosuvastatin = no AND Atorvastatin = NO | Patients using statins need to be evaluated for liver's enzymes level and CK before treatment and at 3 and 12 months after.<br><br>People with LDL ≥ 190 mg / dL or triglyceride ≥ 500 mg / dL must be evaluated for secondary causes of hyperlipidemia, such as hypothyroidism |
| 2. | The association of Angiotensin inhibitors AND angiotensin II blockers is not recommended for hypertension control. | (CAPTOPRIL=YES OR ENALAPRIL=YES OR RAMIPRIL=YES) AND (LOSARTAN=YES OR CANDESARTAN=YES OR VALSARTAN=YES) AND Heart failure=NO AND Ejection fraction <50%=NO | These drugs association is not recommended due to little gain in blood pressure control and the risk of collateral effects, such as hyperkalemia. |
